# Improving Indoor Environmental Quality for Public Health: Impediments and Policy Recommendations

**DOI:** 10.1289/ehp.8986

**Published:** 2007-01-25

**Authors:** Felicia Wu, David Jacobs, Clifford Mitchell, David Miller, Meryl H. Karol

**Affiliations:** 1 University of Pittsburgh, Pittsburgh, Pennsylvania; 2 National Center for Healthy Housing, Washington, DC, USA; 3 Johns Hopkins University, Baltimore, Maryland, USA; 4 Carleton University, Ottawa, Ontario, Canada

**Keywords:** impediments, indoor environmental quality (IEQ), policy recommendations, public education, public health risk

## Abstract

**Background:**

People in modern societies spend more than 90% of their time indoors. Hence, indoor environmental quality (IEQ) has a significant impact on public health. In this article we describe health risks associated with indoor environments, illuminate barriers to overcoming these risks, and provide policy recommendations to achieve healthier indoor environments.

**Objectives:**

The weight of evidence suggests that indoor environmental contaminants pose significant public health risks, particularly among children and the poor, and the societal costs of illnesses related to indoor environments are considerable. Despite the evidence of harm to human health, poor indoor environments are generally difficult to regulate and not of sufficient concern to the general public. We discuss several reasons for this lack of concern about IEQ, focusing specifically on home environments.

**Discussion:**

Economics plays a large role both in political inaction and individual-level indifference. Because little effort has been made to quantify the value of the societal and individual costs of poor housing quality, as well as the benefits achievable by simple interventions, policymakers lack motivation to act on IEQ. Similarly, individual homeowners lack the incentive to remediate homes, as other problems may be more pressing than home environmental quality.

**Conclusions:**

Although the problem of IEQ involves multiple stakeholders and multiple levels of governance, it is possible to establish economic incentives that would set the wheels in motion for action at all levels to achieve healthy home environments. Also important are education and information dissemination on the public health risks associated with indoor environments. These recommendations are intended for all decision makers who have an influence in developing policy to improve indoor environmental quality.

For many centuries doctors and public health workers have understood the role of buildings in causing or exacerbating human diseases. As early as several centuries b.c., the famous Greek “father of medicine” Hippocrates was aware of the adverse effects of polluted air in crowded cities and mines, and Biblical Israelites understood the dangers of living in damp housing. The famed 19th-century nurse Florence Nightingale was cognizant of the connection between health and the dwellings of the population. A comprehensive presentation of the history of understanding the health risks of indoor air is available by Sundell (2004).

Poor indoor environmental quality (IEQ) is an important public health risk worldwide. People in modern societies spend more than 90% of their time in indoor environments (Leech et al. 2002). Most of that time is spent in private homes; in the United States, Canada, and Germany, residence times in the home are very similar, ranging from 15.5 to 15.7 hr/day (Brasche and Bischoff 2005). Hence, indoor environmental quality in households has a significant impact on public health and well-being.

Hazards in indoor environments include biological and chemical contaminants, as well as poor ergonomics, lighting, and physical design. These hazards cause and exacerbate a variety of adverse health effects in humans, ranging from asthma to sick building syndrome to cancer.

Indoor environments encompass homes and other residences, workplaces, transportation vehicles (including cars, trains, planes) and other diverse enclosed settings where individuals spend a portion of their day. Because of the diverse nature of these environments, this article focuses on home environments and presents the public health risks and policy impediments associated with poor IEQ in homes.

There are important barriers to developing policies to improve indoor home environments. Governments are finding it difficult to develop regulations concerning the indoor home environments because these regulations would affect the sacred privacy of individual homes. Because of a scarcity of reliable bio-markers for indoor exposure (with the notable exception of lead), hard economic numbers to indicate the impact of poor housing quality, or widespread citizens’ grassroots efforts to effect changes, there is little policy motivation to achieve healthy indoor environments. Finally, there are socioeconomic challenges. The stakeholder groups that can ensure better IEQ have little motivation to do so, whereas those who live in unhealthy homes often have few means to improve their situation. To achieve improved indoor environmental quality, policy recommendations must include appropriate incentives for multiple stakeholders.

In this article we describe the indoor environmental risks in homes. We then discuss relevant policy issues in the United States and barriers to reducing IEQ risks, both through the marketplace and through public policy. Finally, we provide policy recommendations on how to overcome these barriers to achieve healthier home environments.

## The Public Health Risk of Poor Indoor Environmental Quality

### Hazards in home environments

Indoor home environments are the sites of a variety of biological, chemical, and other environmental hazards. Biological hazards include infectious agents such as bacteria and viruses, molds, endotoxins, and antigens from house dust mites, rodents, cockroaches, pollen, and animal dander. The allergenic constituents of indoor air are predominantly biologic in origin. In recent years, the dramatically increasing rate of asthma in modern societies, coupled with the growing concern regarding indoor environments, has prompted a number of studies concerning exposure to airborne biologic agents and asthma [Institute of Medicine (IOM) 2000].

Chemical hazards include environmental tobacco smoke (ETS), nitrogen and sulfur oxides, ozone, particulate matter (PM), volatile organic compounds (VOCs), pesticides, formaldehyde, and plasticizers (IOM 2000). Exposures to these agents are influenced by chemicals used in building materials, furniture, and other household items; everyday practices such as heating, cooking, cleaning, and home repair; and spontaneous chemical reactions in the indoor environment.

ETS is the largest contributor to suspended PM and respirable sulfates and particles in indoor air. Passive smoking has been shown to be harmful to the health of children. During the first year of life, children exposed to environmental tobacco smoke have higher rates of lower respiratory illness, higher rates of middle ear effusion, and higher rates of sudden infant death syndrome. In addition, children with asthma whose parents smoke have more severe symptoms and more frequent exacerbations (IOM 2000).

A 2000 IOM report (IOM 2000) has associated environmental asthma primarily with indoor as opposed to outdoor exposures. In particular, the IOM committee found sufficient evidence of a causal relationship between asthma exacerbation and exposure to allergens from cats, cockroach, ETS, and house dust mite, and sufficient evidence of association between asthma exacerbation and exposure to allergens from dogs, mold, nitrogen oxides, and rhinovirus. Indoor dampness and mold are associated with upper respiratory tract symptoms, cough, wheeze, and asthma symptoms in sensitized asthmatic persons (IOM 2004). Cancer is also a serious risk associated with exposure to certain indoor air contaminants. ETS and radon exposure are linked with incidence of human lung cancer.

Physical hazards in indoor environments account for many acute as well as chronic injuries. Among such hazards are noise, poor ergonomic settings, and design or decoration that is likely to cause injury (Lyons et al. 2003). Through circadian and endocrine interactions, there may be adverse health effects related to lighting (Schernhammer and Schulmeister 2004). In this present article, we focus primarily on hazardous pollutants found in indoor air in homes.

**Figure 1:**
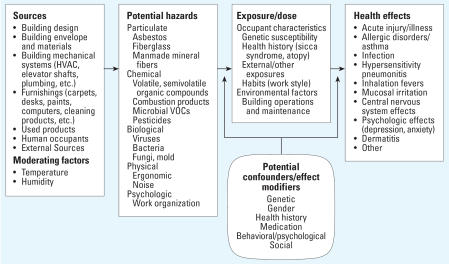
Pathway from built environment to health effects (adapted from Mitchell CS, Hodgson MJ, unpublished data).

Figure 1 illustrates the complexity of the relationship between the indoor structure, the hazards generated as a consequence, and the ultimate health effects in the population. Characteristics of the building structure, such as its composition, contents, and building systems, as well as attributes of the population and activities within the building, all contribute to the health of the indoor environment. Ultimately this can lead to a variety of adverse health effects including respiratory, neurologic, dermatologic, among others, as shown in Figure 1.

### Exposure to and prevalence of hazards in home environments

Human exposures to indoor air hazards are a function of many factors including building characteristics, lifestyles and behaviors, and availability of information and means to remediate known indoor problems. The impact of the regional climate on the types of contaminants encountered must also be considered. For example, house dust mites are common in temperate and humid regions worldwide, as are cockroaches, which are particularly a problem in urban environments and near food sources. Fungi and endotoxins are ubiquitous (IOM 2000).

Indoor carbon monoxide (CO) and nitrogen dioxide (NO_2_) exposures occur whenever high-temperature combustion occurs; indoor sources include gas stoves, space and kerosene heaters, and poorly vented furnaces and fireplaces. Roughly half of U.S. households use gas appliances, and the proportion is much higher in urban areas. About 84% of U.S. households use pesticides in the home. VOC exposure is primarily through indoor environments, with average values ranging from 2 to 84 μg/m^3^ (IOM 2000).

In U.S. households, 27, 31, and 5% have cats, dogs, and birds, respectively. Animal dander can cause asthma and other allergic reactions. By contrast, relatively few households are exposed to allergens from farm animals such as cows, horses, or pigs. The quantities of dust mite allergen found in the air of U.S. houses range from < 0.2 to > 100 ng/m^3^ (IOM 2000).

Moisture and mold are prevalent problems in homes worldwide. Although indoor dampness alone is not a health hazard, it is a precursor to a variety of health hazards more common in moist environments: mold, cockroach, house dust mite, rodents, and off-gassing of chemicals in home surfaces (IOM 2004). In the United States, a questionnaire administered to the homes of 6,273 school children in six cities (Brunekreef et al. 1989) revealed that up to 58% of homeowners reported water in the basement, water damage to the building, or mold or mildew on any surface in their homes. Takaro et al. (2004) found that among 274 Seattle, Washington, homes where children with asthma resided, roughly 44% contained documented visible mold. In a survey of over 16,000 homes throughout Europe, Australia, India, New Zealand, and the United States, 22% of homeowners reported mold or mildew problems within the last year of their time at home (Zock et al. 2002).

Two subpopulations particularly vulnerable to indoor contaminants are low-income persons and children. Indeed, one important barrier to preventing or reducing poor health caused by indoor dampness is poverty (IOM 2004). Census statistics indicate that the poor are more than 3 times as likely (22% vs. 7%) to have substandard-quality housing and that blacks and low-income people are more likely than the general population to be in housing that has extreme physical problems (Evans and Kantrowitz 2002). Lower socioeconomic status has been clearly linked with increased levels of asthma morbidity and mortality (IOM 2000).

Infants and children are at much higher risk of exposure to environmental stressors and toxicants because, pound for pound, they inhale twice as much air at rest, eat 3–4 times as much food, drink 4 times as much water, and have 3 times the rate of skin absorption compared with adults (Bearer 1995). The total annual cost for environmentally attributable childhood diseases in the United States from lead poisoning, asthma, and cancer alone is US$54.9 billion (Landrigan et al. 2002). Although not all of this is due to indoor exposures, their contribution is significant.

Because indoor environmental contaminants are significant public health risks, particularly among children and the poor, both the adverse health effects and exposure levels deserve public policy attention, as the costs to society associated with illnesses related to indoor environments are considerable.

## Why Are IEQ Problems Difficult to Solve?

Despite the evidence of harm to human health, poor indoor environments are generally difficult to regulate and not of sufficient concern to the general public. There are several reasons for this. Economics plays a large role both in political inaction and in the indifference of individuals. Because few efforts have been made thus far to place a monetary value on the societal and individual costs of poor housing quality, as well as the monetary value of benefits achievable by simple and affordable interventions, policymakers lack a motivation to act on IEQ. Similarly, individual homeowners lack incentives to remediate homes, as other problems may be more pressing than home environmental quality.

**Figure 2:**
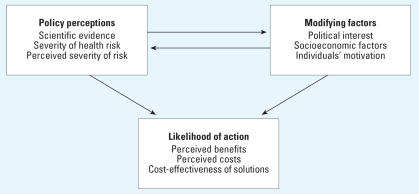
Pathway from perception of IEQ risk and modifying factors to likelihood of political action [adapted from Becker (1974)].

We present a conceptual model that relates the health risks of indoor environmental hazards and modifying factors such as socioeconomic situations with the likelihood of action taken by policymakers to improve IEQ. The model, depicted in Figure 2, is based on the Health Belief Model (Becker 1974), one of the most widely used conceptual frameworks in health behavior research. The model explains why individuals respond (or do not respond) to mitigate their health risks, particularly with respect to adherence to medical regimens. We have applied this model to policy decision makers, who make decisions about whether a particular health risk is worthy of policy attention at the level of legislation, guidelines, regulations, or other forms of action. Figure 2 shows the relationship between three categories of concepts: policy perceptions, modifying factors, and likelihood of action, in which the first two influence the last. In the first section of this article, we discussed the first category: the severity of the health risk. Now we address the other categories to propose why policy action has been lacking in the area of IEQ and how action could be achieved through various recommended strategies.

### Modifying factors

#### Political interest and barriers

Several political barriers exist that prevent development of interest in IEQ regulations. First, in the United States as well as most modern societies, it is very difficult to attempt to regulate private homes as opposed to public buildings such as restaurants or malls. The belief that “a man’s home is his castle” makes it difficult for a government agency to set standards on household behaviors (including product purchases, presence of animals, and cooking, heating, and cleaning habits) that affect indoor air quality.

Second, there has been insufficient political interest in the topic. By contrast, outdoor air became a concern in the United States as a result of a combination of specific incidents and a wave of grassroots action. The Cuyahoga River Fire of 1969 in Cleveland, Ohio, caused by oil and debris on the river from nearby industrial emissions, led to the establishment of “Fresh Air Funds,” charities specifically designated toward improving outdoor air quality. Outdoor air was a focus of Earth Day 1970 and the establishment of the U.S. Environmental

Protection Agency (EPA). Specifically, the Clean Air Act (1970) mentions the need to protect “shared commons.” Indoor air quality protection does not have these same motivators. Unlike outdoor air, there has been no specific “galvanizing moment.” There is no clear-cut “villain,” no public outrage, and thus no grass-roots effort to effect changes in indoor environments. Housing and other indoor spaces have not been perceived to be a “shared commons,” despite that most people live in structures built by and occupied both in the present and the past by others.

Third, the nature of the regulatory framework is more complicated for indoor than for outdoor air. Outdoor air pollution has been regulated by the control of large point source polluters and motor vehicle manufacturers. IEQ, with its many contributing factors and complex interactions, is much more difficult to regulate. Further, many housing code regulations are established locally and not federally.

A final challenge is that scientific data are needed in most cases to establish appropriate guidelines, but finding such data for many indoor pollutants is difficult. The lack of biomarkers of exposure for many contaminants makes setting scientific standards difficult. Current science is still in its infancy in the indoor environment/healthy homes area because these questions largely have been overlooked with scant resources committed to policy-relevant research. There is also a relative lack of economic analysis regarding the impact of diseases and lost productivity associated with poor indoor home environments. Without such compelling statistics, there is no political motivation to develop new regulations on IEQ. When policies have been based on scientific findings, the substantial benefits of making such investments have become more transparent and progress adopting them as standard operating costs has been possible. But in cases where science is wanting, we often lack standards that homeowners, building managers, and governments will implement with confidence, especially for low-income housing that often poses the greatest health risks. Thus, in both the scientific and regulatory dimensions, more information is needed to make IEQ recognized as a politically important problem, although in many cases there is sufficient knowledge to compel action.

#### Socioeconomic factors

The costs of not creating healthy houses, buildings, and communities are rarely identified or understood. These costs are real but are often overlooked or ignored because they are shifted to the health care sector of the economy, where they appear as more expensive medical care (Jacobs 2005). Consequently, investments in healthy homes are unlike other home improvements because they are not reflected in the market price of the structure. That makes health investments appear to be unwise on the part of the homeowner because unlike other home improvement or maintenance investments, they cannot be recovered when the house is sold.

Until health investments are identified in the economic value of buildings, integrating health into routine maintenance, finance, regulatory, and rehabilitation systems will continue to pose a policy challenge for all levels of government. Consequently, the health aspects of housing and indoor environments are generally an afterthought at best, and at worst appear as a burden on affordability or as an “extra” first cost.

Who should pay for healthy home and indoor environment improvements and how can housing and building market forces (both public and private) accommodate the public and environmental health improvements needed? For example, if the value of a house is too low, is additional investment to promote health even possible? How can cost shifting be prevented between the health and housing sectors of the economy? Even in the case of lead-safe housing and radon control in the United States, the value of such health investments in housing is still not routinely reflected in the market price. Without a clear financial return on their individual investments, property owners (rental and home) will be unlikely to incorporate healthy home principles into their construction, rehabilitation, maintenance, and financing decisions (Jacobs 2005). One challenge is to make transparent the costs of not making homes, buildings, and schools healthy and to identify how consumers and building owners can clearly identify homes as being healthy or unhealthy.

#### Individual motivation

The third modifying factor in the model is the human dimension. How do people make decisions, and how can they be motivated to care about their indoor environments? What impediments obstruct their decision making in achieveing healthier home environments? As described above, economics and information dissemination play important roles.

Clearly, income and housing affordability have important health implications. In the United States, 13% of the nation’s lowest income families spend > 50% of their incomes on housing. Nearly one-third of all households in the United States spend 30% or more of their incomes on housing. As a result of widespread affordability problems, crowding is on the increase, some 2.5–3.5 million people are homeless at some point in a given year, and nearly 2 million households live in severely inadequate housing (Harvard Joint Center for Housing Studies 2004). One example of the many adverse health effects of homelessness, a study of homeless children in New York City found that 61% had not been immunized, 38% had asthma, and they had a 50% greater chance of ear infections (Redlener and Johnson 1999).

Despite the obvious benefits that could be achieved through improving indoor environmental quality, a number of barriers exist that relate to individual motivation. Two of the most significant barriers to improving health through reduced indoor dampness are economic in nature: poverty and the uneven distribution of benefits and costs. Low-income families are more likely to be living in substandard housing that has severe physical problems. These families disproportionately lack access to information about dampness-related problems and how to manage them, and even if made aware of them, they often lack the means to address such problems. The uneven distribution of benefits and costs is a barrier because stakeholders crucial to solving indoor dampness problems often lack the economic incentives to do so. They—the housing product manufacturers, builders, engineers, architects, landlords, and maintenance staff—do not usually physically live in the spaces that have been affected by the consequences of their choices. Hence, they may lack the incentive or the proper guidelines to design and maintain buildings to keep out dampness or to collaborate on improving building structures to ensure moisture prevention.

Lack of information and lack of access to information are also important problems. Often, people do not know where to turn if they experience problems with their indoor environments and which information sources they can trust. People may be unaware of the link between their indoor environments and their adverse health symptoms or diseases. Public education on IEQ is severely lacking. Indoor environments are typically not included as a topic in medical care curricula.

## Policy Recommendations to Achieve Healthy Home Environments

Just as the impediments to solving IEQ problems are both economic and information-related in nature, so are the potential solutions to overcoming these problems dealing with economic incentives and modes of communicating information. These must happen both at the levels of policymakers and broader stakeholder groups concerned with housing as well as the individual homeowners. We propose recommendations that will enable stakeholders to better understand the benefits and costs of improving home environments as well as the cost-effectiveness of various interventions. Providing economic incentives and disseminating information are key.

If health benefits from remediation of indoor environments can be translated into economic terms, political action is likely to follow. Otherwise health effects are considered an externality to housing and building policies, and are too often ignored, resulting in inefficient cost shifting. Cost–benefit analyses should be conducted on IEQ; specifically, they should contain information on the following:

- direct and indirect costs of human illness associated with poor indoor environmental quality;
- costs of interventions to achieve healthier indoor environments; and
- direct and indirect monetized benefits associated with healthier indoor environments.

An example concerns remediation of U.S. homes with lead-based paint and the impacts on children’s health. Because of careful legislation, regulation, education, research, and enforcement, children’s blood lead levels have decreased dramatically since the 1970s. The U.S. Department of Housing and Urban Development (HUD) and the U.S. EPA have estimated that from 2000 to 2010 the cost of reducing blood lead levels from exposure to lead-based paint will be $2.4 billion over 10 years (HUD and U.S. EPA 2000). On the other hand, a retrospective analysis showed that annual monetized benefits, considering a 2.2–4.7 point increase in IQ per child affected, are $110 billion–$319 billion (Grosse et al. 2002). In short, the monetized benefits greatly exceed the costs. It will be necessary to document, as with the case of lead remediation improving children’s IQ, that the benefits of home remediation to reduce exposures to other contaminants will multiply throughout society.

One example of a common housing intervention that likely has unrecognized multiple health benefits is window replacement. The cost of replacing old windows with energy-efficient ones averages $6,000 per housing unit, which is approximately equal to the increase in housing value due to fuel savings and appearance alone (Nevin 2000). But window replacement has also emerged as a way of controlling lead dust and lead paint hazards (National Center for Health Housing 2004). The health benefit of such window replacement is estimated to be over $20,000 per housing unit (HUD 1999) due to avoided childhood lead poisoning. The health benefits associated with lead poisoning prevention, when added to the increase in home value and energy savings, clearly dwarf the expense of new windows. The economic benefits of window replacement, which includes other health improvements such as reduction of moisture and mold to reduce asthma risk, have not yet been quantified. But the common view is that the investment in new windows will only occur if the value of the property increases so that the investment can be recovered when the property is sold. Governments should consider adopting policies that uncover the health consequences of building investments (such as window replacement) rather than keeping such information hidden.

Multiple stakeholders could be given economic incentives to ensure healthy housing; for example, a reward or special recognition (such as a label) for meeting a certain level of quality. In the U.S. energy conservation arena, such means currently exist and may be expanded to cover health-related issues as well. The U.S. EPA Energy Star label is one way that consumers can determine if windows, appliances, and other products meet government standards for energy conservation (U.S. EPA 2007a). This is a voluntary program that manufacturers can choose to join if their products meet certain standards. In a similar vein, homes and rental spaces could be awarded a particular label for meeting a certain quality of indoor environment. Landlords also could be given awards and public recognition for providing rental spaces with good IEQ. Systems are emerging that could accomplish this, such as U.S. EPA’s Energy Star Plus Indoor Air and the Enterprise Foundation’s Green Communities program (U.S. EPA 2007b).

In addition to adopting policies that promote such investments, local governments should develop and implement hazard identification and remediation protocols. Although more research is needed to standardize such protocols, one effort to build local capacity is a joint collaboration among HUD, the Centers for Disease Control and Prevention, U.S. EPA, the National Center for Healthy Housing, and a network of training providers to establish the Healthy Homes Training Center and network.

For both Energy Star and Healthy Homes, it is clear that public education and information dissemination are key to achieving healthy home environments. People must understand the health benefits associated with healthy houses in order to have the incentives to purchase them. The questions are “Who should communicate to the public?” and “How can we persuade people to make effective behavioral changes?”

Several pilot studies have shown that educational efforts carried out by community health workers and nurses, in combination with environmental interventions in the home, are effective in changing the indoor environment and motivating behavior change in ways that promote healthier home environments (Carter et al. 2001; Krieger et al. 2005; Morgan et al. 2004). In the successful educational interventions, the strategies taught to home dwellers were usually simple and feasible: cleaning and vacuuming more frequently with HEPA-filter vacuums, increasing fresh air ventilation in the home, putting covers on pillows and mattresses, and noticing and taking steps to prevent excess moisture. Krieger et al. (2005) found that the educational intervention combined with the cost of providing basic cleaning and bedding materials to families was cost-effective, as these measures led to a decrease in the number of hospital visits for children with asthma compared with a case–control study. The cost-effectiveness of other types of home environmental interventions is discussed in Wu and Takaro (2007). On the other hand, there is a need for more studies on the effectiveness of interventions that focus on building operations and maintenance by building owners/operators,

## Discussion

There is now sufficient evidence from a risk assessment standpoint that home environments contain multiple health hazards that deserve public policy attention. However, the problem of IEQ involves multiple stakeholders who lack both the motivation and the proper information necessary to make changes. The combination of inaction at both the individual and societal levels makes healthy indoor environments difficult to achieve. Even if regulatory decision makers, architects, and engineers could be convinced to provide high-quality homes to all, indoor environments could still be poor if the building dwellers did not know how to take proper care of their homes. Unfortunately, some low-quality homes are virtually impossible to cost-effectively remediate, and the regulatory and legal remedies available to compel these improvements are often lacking. The result in both of these situations is suboptimal human health conditions.

Although the problem involves multiple stakeholders and multiple levels of governance, it is possible to establish economic, social, and other incentives that would initiate action at all levels. Individuals must view their long-term health as an important investment when making decisions to purchase and maintain a home, even if this factor is not necessarily reflected in the price of the home. Regulatory decision makers also need comprehensive economic analyses on the costs associated with poor indoor environments and the benefits of healthy ones in order to gain the political means to effect broad changes in IEQ. Ultimately, the housing market and housing providers must include health-related investments in market prices and the costs of doing business to stimulate further investment and action.

Education and information dissemination on the public health risks associated with indoor environments are essential. Policy-makers must be well informed of such risks in order to make useful public health decisions. Similarly, individuals must understand both the health consequences of poor indoor environmental quality, and some simple and feasible interventions to improve IEQ. Public health education conducted by community health workers has been found to be cost-effective regarding behavioral changes in the home and resulting health benefits.

Indeed, policy changes at multiple levels are needed to achieve healthy indoor environments. It is quite certain that the benefits of such investments, measured in terms of improved human health and productivity, significantly outweigh the costs.
